# Mechanical Performance of High-Strength Sustainable Concrete under Fire Incorporating Locally Available Volcanic Ash in Central Harrat Rahat, Saudi Arabia

**DOI:** 10.3390/ma14010021

**Published:** 2020-12-23

**Authors:** Muhammad Nasir Amin, Kaffayatullah Khan

**Affiliations:** Department of Civil and Environmental Engineering, College of Engineering, King Faisal University (KFU), Al-Ahsa 31982, Saudi Arabia; kkhan@kfu.edu.sa

**Keywords:** volcanic ash, high-strength concrete, elevated temperature exposure, residual compressive strength, ultrasonic pulse velocity

## Abstract

This study investigated the effect of elevated temperatures on the mechanical properties of high-strength sustainable concrete incorporating volcanic ash (VA). For comparison, control and reference concrete specimens with fly ash (FA) were also cast along with additional specimens of VA and FA containing electric arc furnace slag (EAFS). Before thermal exposure, initial tests were performed to evaluate the mechanical properties (compressive strength, tensile strength, and elastic modulus) of cylindrical concrete specimens with aging. Additionally, 91 day moist-cured concrete specimens, after measuring their initial weight and ultrasonic pulse velocity (UPV), were exposed up to 800 °C and cooled to air temperature. Subsequently, the weight loss, residual UPV, and mechanical properties of concrete were measured with respect to exposure temperature. For all concrete specimens, test results demonstrated a higher loss of weight, UPV, and other mechanical properties under exposure to higher elevated temperature. Moreover, all the results of concrete specimens incorporating VA were observed before and after exposure to elevated temperature as either comparable to or slightly better than those of control and reference concrete with FA. According to the experimental results, a correlation was developed between residual UPV and residual compressive strength (RCS), which can be used to assess the RCS of fire-damaged concrete (up to 800 °C) incorporating VA and EAFS.

## 1. Introduction

Concrete is one of the most widely used construction materials on the face of Earth. Although concrete exhibits outstanding engineering performance in different extreme environments, it faces a significant challenge when exposed to fire due to improper design or as a result of an accident. Concrete is considered a semi-permanent material that neither burns nor emits toxic gases or smokes. Resistance to elevated temperatures and fire is one of the essential properties of concrete for maintaining its structural integrity [1]. However, intense and prolonged exposure to fire can significantly deteriorate its structural performance [2,3,4,5]. Fire causes an abrupt increase in temperature in a relatively short duration, which results in the degradation of the physical, chemical, and mechanical properties of concrete. The elevated temperature significantly influences the color, shape, stiffness, and strength of concrete structures. The loss of strength and elastic properties is generally associated with cracking and spalling of concrete, which is caused by the thermal gradients in concrete mass [6,7]. Deterioration in the performance of concrete due to exposure to fire is attributed to the structural change in the cement matrix at the microscopic level. The hydration products of the cement matrix decompose when subjected to elevated temperatures. Moreover, the thermal incompatibility of concrete elements and vapor pressure development further cause degradation in the durability of concrete. Over the last few decades, extensive research has been conducted to assess the structural performance of concrete subjected to high temperatures, and several techniques have been utilized to cope with the elevated temperatures. Different natural and synthetic materials have been added to concrete to improve its fire resistance properties. Among these, pozzolanic materials have shown a good tendency of better performance in enhancing the fire-resisting capacity of concrete [2].

In recent years, extensive research was carried on the potential use of naturally available materials and waste generated from various industrial and agriculture sectors in the production of high-strength and high-performance sustainable concrete [8,9,10]. The pozzolanic ashes obtained from different processes in industries have shown promising results when incorporated in mortar and concrete. The usage of such pozzolanic materials is gaining popularity over time due to its superior structural characteristics, eco-friendly nature, and high-energy conservation potential. All these properties collectively make pozzolanic materials one of the major sustainable sources of cement replacement in concrete. The commonly used supplementary cementitious materials are rice husk ash (RHA) [11], silica fume (SF) [12], fly ash (FA) [13], wheat straw ash (WSA) [14], volcanic ash (VA) [15], ground granulated blast furnace slag (GGBFS) [16], and metakaolin (MK) [17]. The addition of these pozzolanic materials to concrete improves its mechanical properties and enhances the durability. Moreover, it also improves the resistance of concrete to extreme environmental conditions such as freeze and thaw, alkali–silica reaction (ASR), and sulfate and chloride attacks. The chemical and mechanical properties of these secondary cementitious materials (SCMs) have been widely investigated, while some characteristics are still to be explored. One of the vital areas that requires more in-depth knowledge is the exposure of concrete (containing pozzolanic materials) to highly elevated temperatures.

Various SCMs were evaluated against high temperatures including FA, VA, and GGBFS. Nasser and Marzouk conducted one of the first studies on FA-blended mass concrete exposed to elevated temperature in 1979 [18]. They observed an increase in strength at temperatures between 121 and 149 °C, whereas a 60% and 65% reduction in strength and elasticity was recorded at the elevated temperatures of 177 and 232 °C, respectively. A decrease in the compressive strength of concrete containing 10–40% FA was recorded when it was exposed to a temperature ranging from 200 to 800 °C. Furthermore, cracks were detected at 400 to 800 °C [19]. Khan and Ali [20] investigated the effects of 25% and 40% FA as a replacement of cement on the compressive strength of concrete exposed to high-elevated temperatures under stressed and unstressed conditions. They discovered that the specimen having 40% FA subjected to 400 °C under a stressed condition showed a minimum reduction in the residual compressive strength (RCS) as compared to the control specimens under the same temperature. Nadeem et al. [17] investigated the performance of FA- and MK-blended cement mortar under high temperatures from 27 to 800 °C. A reduction in strength and durability of concrete was noticed beyond 400 °C, which can be considered a critical temperature in terms of durability and strength loss. The 20% FA-mixed mortar showed better results compared to the 20% MK cement mortar. Pathak and Siddique [21] reported that the presence of fly ash in self-consolidating concrete improved its chloride ion penetrability along with enhancing its mechanical properties at elevated temperature. Some studies showed that an increase in the substitution level of FA results in a reduction in the thermal conductivity of concrete. However, the thermal conductivity is also affected by the water-to-binder ratio (w/b). A higher w/b reduced the coefficient of thermal expansion of concrete containing 50% FA, while it increased for lower w/b at the same replacement level [22,23]. The addition of GGBFS as a partial replacement of cement in concrete has proven to be successful in enhancing the mechanical properties of concrete along with an increase in the fire resistance capacity [24]. Wang [25] observed that around 20% replacement of cement with GGBFS improved the fire resistance of cement mortar exposed to high temperatures. Poon et al. [26] studied the influence of SCMs on the strength and durability properties of standard and high-strength concrete (HSC) exposed to elevated temperatures and observed that the specimens containing GGBFS showed better performance compared with control specimens. Demirel and Kelestemur [27] exposed a concrete blended with a binary mixture of finely ground pumice (FGP) and SF to high temperatures. They concluded that the addition of both FGP and SF decreased the compressive strength and reduced the unit weight of concrete at a temperature above 600 °C. Mohamad et al. [28] experimentally explored the performance of VA in cement mortar and they highlighted that the replacement of cement with VA affects the properties of mortar at a later age at both ambient and high temperatures. The addition of VA up to 20% in the HSC exhibited better resistance to chloride penetrability and deterioration along with an enhancement in the residual strength subjected to a high temperature of about 800 °C. However, reduced durability and loss of strength were noticeable when the temperature increased beyond 800 °C [29]. According to Khurram et al. [30], mortar samples with 20% VA as a replacement of cement possessed better mechanical properties at elevated temperature as compared to control samples.

The per capita consumption of cement in the Kingdom of Saudi Arabia (KSA) has been recoded as one of the highest in the world [31]. The cement and concrete industry is responsible for around 10% of the total global CO_2_ emission, which causes global warming and climate change-related issues [32]. Therefore, there is a vast global demand to reduce the use of cement by replacing it with natural pozzolanic/cementitious materials or industrial byproducts to reduce its carbon footprint [33]. Currently, KSA is importing different types of commercially available SCMs such as FA, GGBFS, and SF to fulfil their fast-growing concrete industry demands, which is obviously a burden on the kingdom’s economy.

Like many other industry byproducts (FA, GGBFS, SF), VA is available in abundance around the globe, particularly in areas having active volcanos [34,35,36,37,38]. Its widespread use as a building material, especially in cement and concrete, has shown promising results. Similar to other parts of the world, huge reserves of natural pozzolanic materials, commonly known as volcanic scoria or VA, are also present in the western and northwestern region of Saudi Arabia [39,40]. Many researchers investigated the potential use of locally available VA as a partial substitute of cement in mortar and concrete [31,41]. The results of past research studies showed outstanding potential of substituting expansive commercially available pozzolans with locally available VA. The promising results of past studies are an indicator to further explore the locally available VA. This is inevitable to meet the future infrastructural demands of KSA and to achieve its goals of environmental sustainability and economy. Although, promising results after replacing cement with locally available VA (up to 20%) have been reported in terms of achieving better mechanical and durability properties of both mortar and concrete at later ages, for its broader applications in concrete, there is a need to perform extensive research to evaluate its performance under extreme loading scenarios such as a fire. Therefore, this study aimed to assess the effect of aging and elevated temperatures on the mechanical properties (compressive strength, tensile strength, and elastic modulus) of a binary HSC containing 20% VA and 20% FA as a partial substitute of cement. Furthermore, the effects of aging and elevated temperatures on the mechanical properties of a ternary HSC containing 20% VA with 10% EAFS and 20% FA with 10% EAFS were also studied. In addition to mechanical properties, the effect of elevated temperatures on the weight loss of concrete samples and the changes in their ultrasonic pulse velocity (UPV) were also investigated. A comparison of the results of HSC containing VA (before and after elevated temperature exposure) to those of control concrete (CC) and reference FA-based HSC is presented. In order to assess the RCS of fire-damaged concrete, an effective correlation between RCS and residual UPV of concrete containing VA and EAFS was developed. Lastly, the validity of the proposed equation was discussed and compared with other published works.

## 2. Materials and Methods

### 2.1. Materials

**Figure 1:**
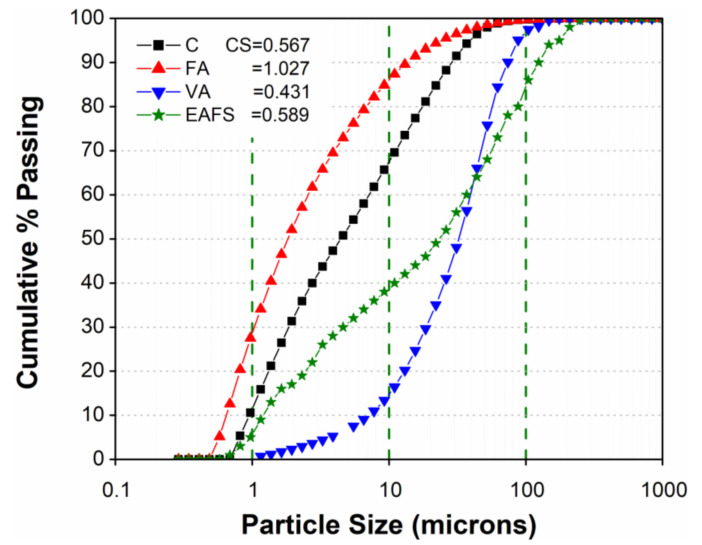
Particle size analysis curve of cement, volcanic ash, fly ash, and electric arc furnace slag.

**Figure 2:**
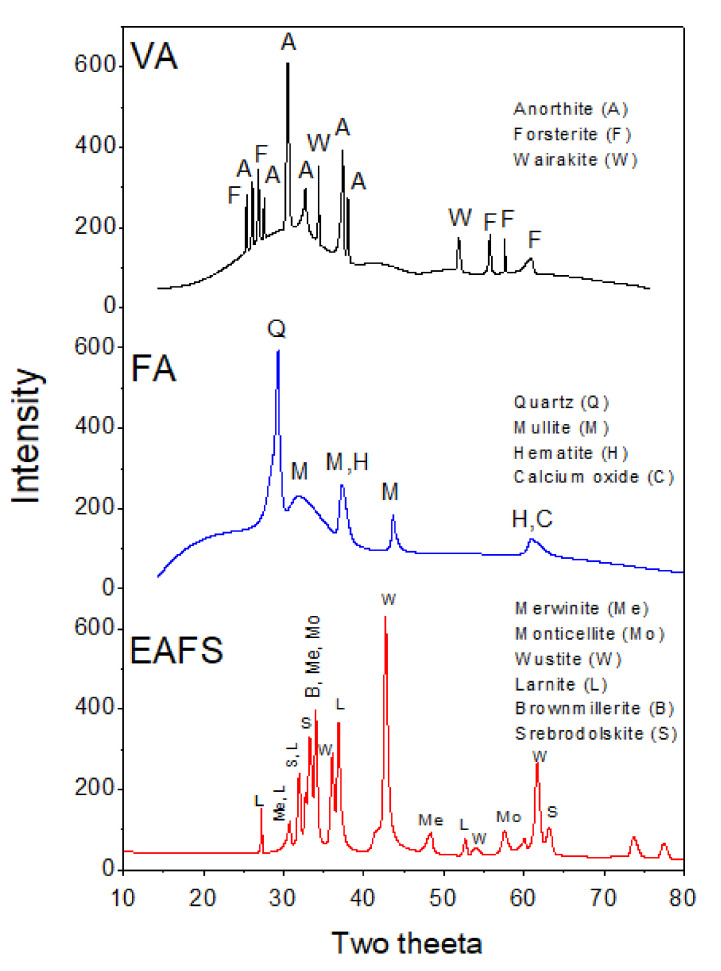
X-ray diffraction analysis of volcanic ash, fly ash, and electric arc furnace slag.

Materials used in this study were ordinary Portland cement (C) from Saudi cement factory, commercially available FA (Class F), EAFS from Saudi Basic Industries Corporation (SABIC), Saudi Arabia, and locally available natural VA from Supper Burkanni Block Co. Jeddah, Saudi Arabia with an average particle size of 30 µm. The EAFS was obtained from SABIC in the form of small-size aggregates (up to 5 mm) and ground in the laboratory to achieve the desired fineness. The chemical composition of all the materials used was obtained by performing X-ray fluorescence (XRF) analysis as shown in Table 1. The particle size analysis for all the materials was performed using laser diffraction with a Microtrac S3500, and the particle analysis curves are shown in Figure 1. The corresponding D10, D50, and D90 values for all the materials used in this study are also shown in Table 2. X-ray diffraction (XRD) analysis was also performed using a Rigaku MiniFlex II on fine powder samples of FA, EAFS, and VA. The main mineral phases present in these materials are shown in Figure 2.

According to ASTM C618-15 [42], the minimum sum of percentages of silicon dioxide (SiO_2_), aluminum oxide (Al_2_O_3_), and iron oxide (Fe_2_O_3_) is 70% for both Class-N and Class-F pozzolanas. As demonstrated by the chemical analysis results (Table 1), the sum of SiO_2_, Al_2_O_3_, and Fe_2_O_3_ for both VA (Class-N) and FA (Class-F) was found to be 73.6% and 84.7%, respectively, which indicated the pozzolanic nature of both VA and FA. A more detailed investigation on the pozzolanic nature and reactivity of both VA and FA used in this study can be found in another study by Khan et al. [15]. Unlike other materials, EAFS, due to its high fineness, was just used as a filler in this study.

**Figure 3:**
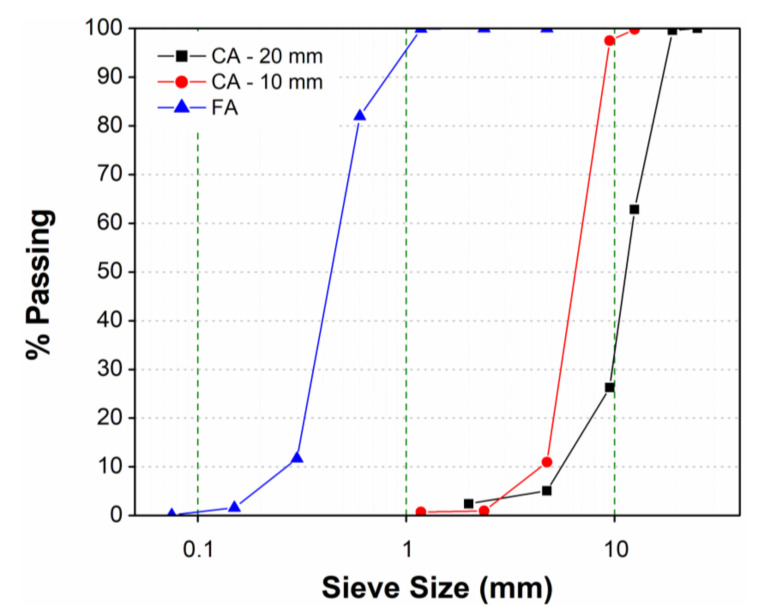
Particle size distribution of fine and coarse aggregates.

Locally available hard limestone-based aggregate and desert sand were used in the preparation of high-strength concrete mixtures. The physical properties of both coarse and fine aggregates were tested according to the ASTM standards, as shown in Table 3. Two different sizes of coarse aggregates with a maximum particle size of 20 mm and 10 mm were used for preparation of the concrete mixture. Sieve analyses of fine aggregate and both sizes of coarse aggregates were determined according to ASTM C136 [43], as shown in Figure 3. The specific gravity and water absorption of coarse aggregates were determined according to ASTM C127 [44]. The fineness modulus of desert sand was determined according to ASTM C33 [45]. The specific gravity and water absorption of fine aggregate were also determined according to ASTM C128 [46].

#### Grinding of EAFS

A PULVERISWTTE 5/4 planetary mill classic line with four grinding bowl fasteners was used to grind the EAFS to change its physical state from small-size aggregates to powder form. The machine consists of four grinding bowls each with 250 mL capacity, made from hard metal tungsten carbide, with steel casing. Each bowl consists of five grinding balls (diameter 30 mm), made from hard metal tungsten carbide. Each bowl can grind up to 125 ± 1 g of material, which means that the planetary mill used in this study has the potential to grind up to 500 g of EAFS in one complete cycle. A measured quantity of 125 g of EAFS aggregates was poured into each bowl of the grinding mill and subjected to grinding for 30 min at a fixed rotation speed of 300 rpm. The grinding was performed in three consecutive cycles such that each cycle consisted of 10 min of grinding followed by a 10 min pause to lower the temperature of the grinding bowl. Each grinding cycle lasted for a total of 50 min. Finally, after completion of the grinding process, the ground EAFS after removing from the bowls was stored in sealed plastic bags to prevent any potential moisture transportation.

### 2.2. Mix Proportions and Specimen Preparation

#### 2.2.1. Mix Proportions

As shown in Table 4, five different HSC mix proportions including a control mix were used in this study. The mixture of control concrete (CC) contained 100% cement, while the remaining four mixes contained pozzolanic materials as a partial substitute of cement, with and without EAFS. These concrete mixtures having pozzolanic materials were identified as binary and ternary mixes. For instance, the two binary mixes incorporating 20% VA and 20% FA as a partial substitute of cement were identified as V20 and F20, respectively. The two ternary mixes containing 10% EAFS as additive/filler with V20 and F20 were identified as V20S10 and F20S10, respectively. The purpose of adding 10% ground EAFS to ternary mixtures was to evaluate its effectiveness in improving the fire resistance of concrete as compared to CC and binary mixes. Moreover, the purpose of studying two different types of binary and ternary concrete mixes of VA (V20 and V20S10) was also to determine the best possible cement replacement blend without compromising strength. However, the binary and ternary mixes with FA were selected as a reference to investigate if the naturally occurring VA or its blend with locally available EAFS can be a viable alternative to this popular pozzolanic material. To produce HSC, a constant water-to-binder ratio of 0.35 was used in all mixes with a slight variation in aggregate-to-binder (a/b) and sand-to-aggregate (s/a) ratios by weight. A naphthalene-based high-range water-reducing admixture was used in different percentages by weight of binder materials to achieve a desired workability. For all mixes, different dosages of superplasticizer were selected such that the slump values fell within 120 ± 30 mm. The target total air content for non-air-entrained concrete mixes having a maximum particle size of coarse aggregate of 20 mm was set to 3.0% ± 0.5%.

The mixture proportions for HSC in this study were prepared following ACI 363.2R-11 [47], according to which HSC is defined as concrete with a specified compressive strength of 55 MPa or greater. However, as discussed in the next section, the curing of concrete specimens continued up to 91 days to achieve the maximum potential strength as required by ACI 211.4R-08 for HSC containing cementitious materials [48]. According to ACI 211.4R-08, as most HSC mixtures use FA, SF, slag cement, or other cementitious materials, HSC can gain considerable strength after the normally specified 28 day age. Therefore, to take advantage of this characteristic, many specifications for the compressive strength of HSC were modified from the typical 28 day criterion to 56 days, 90 days, or later ages.

#### 2.2.2. Mixing and Preparation of Cylindrical Concrete Specimens

All the concrete ingredients were mixed by using a power-driven rotating pan mixer, according to the procedure specified in ASTM C192 [49]. Immediately after mixing, the fresh properties of concrete, such as slump, air content, and unit weight, were measured for all the mixes, as listed in Table 4. Although the slump and total air content for all mixes were found within the target values. However, despite of low dosage of superplasticizer, F20 concrete exhibited the highest workability among all mixes, due to the spherical particles of FA. The relatively low slump values of CC and F20S10 were due to the irregular shaped particles of cement and the use of an additional amount of sand in this particular mix, respectively.

Following the measurement of fresh properties, 42 cylindrical specimens (100 mm diameter × 200 mm height) were fabricated for each mix using steel molds according to ASTM C 39 [50]. All the cylindrical molds after fabrication were covered with a plastic sheet and stored in the concrete mixing laboratory under control environmental temperature and humidity (T = 20 ± 1 °C and RH = 60% ± 5%). The concrete specimens were demolded after 24 h of casting and continuously cured in a water tank until the age of testing (7, 28, and 91 days). After reaching the required curing time, both the top and the bottom ends of all concrete specimens were smoothed using a concrete end surface grinder to perform further tests. For each mix, 18 concrete specimens out of 42 were used to test the development of mechanical properties (compressive strength, elastic modulus, and split tensile strength). To calculate the average result of three identical specimens at each testing age, three identical concrete specimens were reserved for the compressive strength and elastic modulus test and three were reserved for the split tensile strength test. The remaining 24 specimens (continuously moist-cured for 91 days) of each mix were used to test the mechanical properties of concrete after their exposure to four distinct elevated temperatures of 200, 400, 600, and 800 °C. The reason for selecting 91 day cured specimens for exposure to elevated temperatures was to ensure that the concrete gained maximum potential strength as required by ACI 211.4R-08 for HSC containing cementitious materials [48]. To calculate the average result of three identical specimens at each elevated temperature, three concrete specimens were reserved for the compressive strength and elastic modulus test and three were reserved for the split tensile strength test. Furthermore, the same specimens of each mix, which were reserved for the compressive strength and elastic modulus test, were also used to measure the weight loss due to elevated temperature exposure and to perform the UPV test before and after exposure to elevated temperatures.

### 2.3. Test Methods

#### 2.3.1. Thermal Exposure

Before exposure to the elevated temperatures, all the cylindrical specimens were first oven-dried at 100 °C until a constant weight was achieved. The purpose of the prior drying was to remove the capillary water and reduce the risk of potential concrete spalling [51]. Subsequently, the concrete specimens were cooled down to room temperature to measure their individual initial weight and UPV. Afterward, the specimens were placed inside an electric furnace for exposure to the desired elevated temperatures. To maintain the thermal uniformity, three identical specimens of each concrete mix (Table 4) were placed inside the furnace simultaneously.

**Figure 4:**
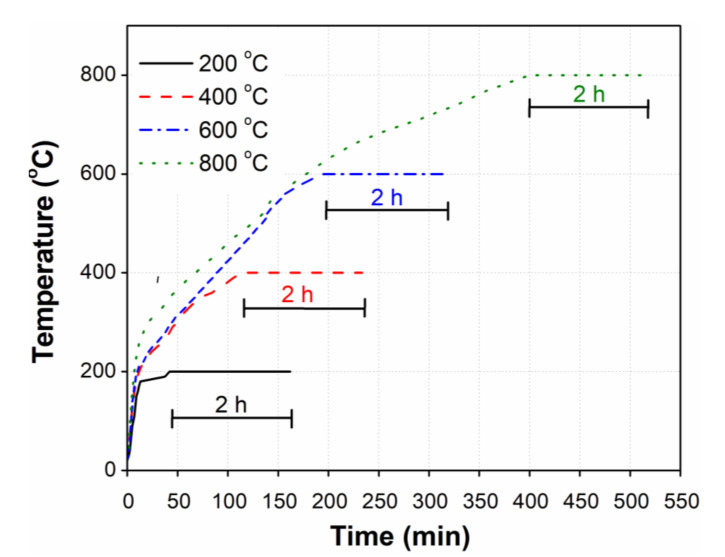
Temperature variation inside the furnace for different elevated temperatures.

The internal temperature of the furnace was set to increase from room temperature to different elevated temperatures (200, 400, 600, and 800 °C). Due to the default variation in the rate of temperature increase in the furnace, the total time required to reach the maximum temperature was different for each elevated temperature. The actual time taken by the furnace to reach the specified elevated temperature (200, 400, 600, and 800 °C) was recorded and is shown in Figure 4. The program was set in the furnace to expose specimens for a period of 2 h after attaining the peak temperature. After the exposure of the specimens to set temperatures, the furnace was automatically turned off and the specimens were allowed to cool inside the furnace. After approximately 24 h, all the concrete specimens were removed from the furnace and their individual weights were measured using a sensitive balance to calculate the weight loss for each. After this, all the specimens were carefully wrapped in a plastic sheet to avoid any rehydration until further tests for residual UPV and mechanical properties.

#### 2.3.2. Weight Loss

First, the initial weight of each cylindrical concrete specimen used in destructive compression tests was measured before its exposure to elevated temperatures. Subsequently, the residual weight of each specimen after exposure to the specified elevated temperature (200, 400, 600, 800 °C) was measured to calculate the weight loss. The weight loss is reported as the ratio of residual weight of specimen after each elevated temperature exposure to its respective initial weight.

Weight loss is commonly related to the loss of moisture present in different forms within concrete mass, such as free, capillary, absorbed, and chemically bound water. The weight loss at different elevated temperatures causes an alteration in the microstructure of cement paste, which directly affects the mechanical and durability properties of concrete. Therefore, the purpose of this test was to evaluate the impact of different elevated temperatures on weight loss of concrete containing locally available natural pozzolana (VA and its blend with EAFS) as compared to CC and reference FA-based concrete (VA and its blend with EAFS).

#### 2.3.3. Compressive Strength, Splitting Tensile Strength, and Elastic Modulus

Mechanical properties such as the compressive strength, elastic modulus, and split tensile strength of all concrete specimens were evaluated before and after exposure to elevated temperatures. At first, tests for mechanical properties on three identical specimens of all mixes (Table 4) were performed before thermal exposure at different ages such as 7, 28, and 91 days. Lastly, additional tests on 91 day cured specimens of all concrete mixes were performed after their exposure to specified elevated temperatures.

The test for compressive strength of cylindrical specimens before and after exposure to elevated temperatures was performed according to ASTM C39 using a 1500 kN unconfined compression test machine (UCT 1500). The loading rate in the UCT was set to 0.2 MPa/s. The split tensile test on the cylindrical specimen was performed according to ASTM C 496 using a 300 kN universal testing machine (UTM) [52]. The pace rate of the UTM crosshead was set to 1 mm/min. The elastic modulus of concrete specimens was calculated from the stress–strain data obtained from compression tests. The strain of each specimen, corresponding to any stress level, was calculated using the average of displacements obtained from two equally spaced linear variable displacement transducers (LVDTs) attached to the compression test specimen. As specified in ASTM C469 [53], the chord modulus of elasticity of the concrete specimen was calculated using the stress values corresponding to 40% of the ultimate load and 50 millionth of longitudinal strain, and the longitudinal strain corresponding to 40% of maximum strength.

#### 2.3.4. Ultrasonic Pulse Velocity

A well-known nondestructive UPV test on three identical concrete specimens of each mix before and after exposure to specified elevated temperatures was performed using a UPV tester, consisting of a transmitting and receiving transducer capable of generating and receiving 50 kHz ultrasonic pulse. To perform the UPV test, transducers were placed on smoothed ends of the concrete specimen after applying silicone gel as required by ASTM C597 for the smooth transfer of signals through concrete specimens [54]. The UPV of tested specimens was calculated in m/s by dividing the distance traveled by the pulse (0.2 m) by the recorded time taken by the pulse to travel that distance.

## 3. Result & Discussion

### 3.1. Weight Loss

**Figure 5:**
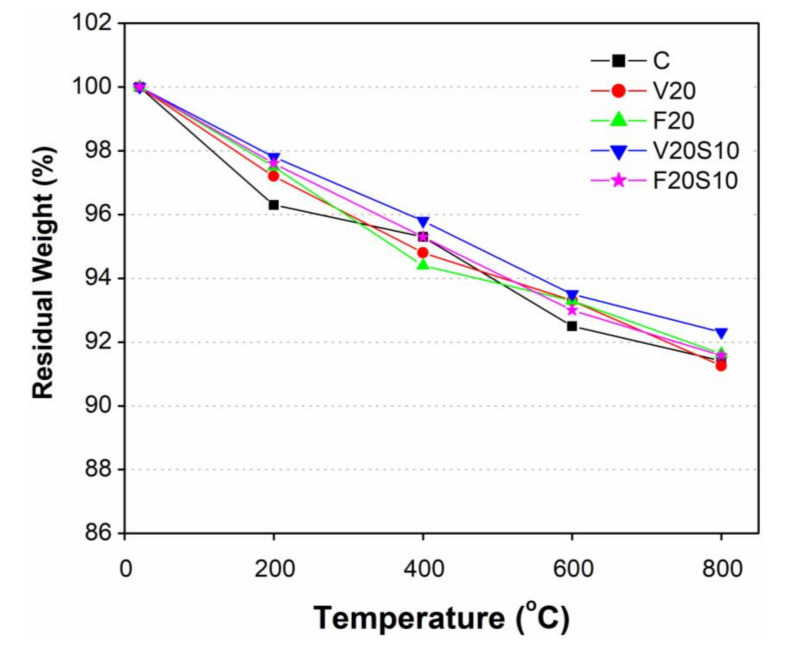
Residual weight of concrete specimens after exposure to different elevated temperatures.

Figure 5 shows the comparison of weight loss among concrete specimens (C, F20, V20, F20S10, and V20S10) subjected to four distinct elevated temperatures, i.e., 200, 400, 600, and 800 °C. As shown, the residual weight (%) along the vertical axis is a measure of percentage weight retained at any particular elevated temperature compared to the corresponding weight of the specimen at room temperature. To measure the weight retained, all the concrete specimens after being exposed to elevated temperatures for a specified duration were gradually cooled in normal air inside the furnace. To study the residual weight of all tested concrete samples, the effect of elevated temperature was divided into four heating phases as 20–200, 200–400, 400–600, and 600–800 °C.

In fact, the weight loss increased with the increase in exposure temperature irrespective of the type of tested concrete sample. In the first heating phase (20–200 °C), the CC specimens showed the highest weight reduction among all (3.7%), whereas the lowest was observed in V20S10 ternary concrete samples (2.2%). The weight loss in this heating phase is due to the moisture evaporation from the sample surface to the atmosphere. Interestingly, the specimens of ternary concretes containing pozzolanic materials with EAFS (V20S10 and F20S10) exhibited relatively lesser weight losses as compared to their corresponding binary concrete specimens without EAFS (V20 and F20). The current results of relatively lesser weight loss of ternary mixes than those of binary mixes are consistent with the findings of past studies on cement mortars [30]. Moreover, the slightly lesser weight loss of concrete samples containing pozzolanic materials than that of CC is attributed to their late pozzolanic reactivity. In the second heating phase, when the temperature raised from 200 to 400 °C, the reduction in weight increased significantly and the highest and minimum weight losses in heating phase 2 were observed in F20 (residual weight 94.4% at 400 °C from 97.5% at 200 °C) and CC specimens (residual weight 95.3% at 400 °C from 96.3% at 200 °C), respectively. This continued weight loss in heating phase 2 could be due to the further evaporation of residual moisture content being retained in a previous heating phase. Similar to heating phase 1, once again the V20S10 concrete specimens showed the highest weight retained (95.8%) among all tested concretes followed by CC (95.3%), F20S10 (95.3%), V20 (94.8%), and F20 (94.4%) concrete specimens. Furthermore, the trend of higher weight retained by ternary concrete specimens as compared to their corresponding binary concrete specimens continued in heating phase 2. However, the impact of EAFS in ternary concretes on weight retained was noticeable in heating phase 2 as compared to heating phase 1. In heating phase 3 (400 to 600 °C), a continued rate of weight reduction was noted in ternary concrete specimens, while, in comparison to heating phase 2, the rate of weight loss in binary concrete specimens (V20 and F20) slightly dropped. Similar to heating phase 1, the highest rate of weight loss, among all tested concrete samples, was again observed in CC specimens (residual weight 92.5% at 600 °C from 95.3% at 400 °C). According to Guo et al. [55], the weight loss in concrete at 600 °C occurred due to the evaporation of water that was produced as a result of decomposition of Ca(OH)_2_ to CaO and H_2_O. In the final heating phase (600 to 800 °C), the rate of weight reduction slightly dropped in CC and in ternary concrete samples, while a slight increase in rate of weight reduction was observed in binary concrete samples as compared to previous heating phase. Lastly, all the tested concrete samples experienced an almost identical net weight loss of 8.6% after heating to 800 °C except for V20S10 concrete samples. The net amount of weight loss in V20S10 concrete samples was lowest at 7.7%. The lowest amount of weight loss in V20S10 concrete could be because of no or lesser retention of water due to the simultaneous presence of VA and EAFS.

Consistent with past studies, the aforementioned weight loss phenomenon in all the tested concrete samples may be attributed to the changes in their stiffness and mechanical properties [56,57]. According to Saha et al. [58], the weight loss under high elevated temperature affects the structural integrity. This is because of the cement paste that loses its binding properties due to the evaporation of binding water from the structure of C-S-H and the decomposition of Ca(OH)_2_.

### 3.2. Mechanical Properties of Concrete before Elevated Temperature Exposure

Before exposing the concrete specimens to elevated temperatures, the development of different mechanical properties of concrete such as compressive strength, tensile strength, and elastic modulus were calculated with aging at 7, 28, and 91 days. In addition, the UPV of concrete was also measured before elevated temperature exposure. The UPV test was conducted on 91 day cured specimens as the concrete mixtures containing pozzolana attain their maximum potential strength at later ages. A summary of test results before elevated temperature exposure is given in Table 5. All the results presented in this table represent the average of three identical specimens. It is worth mentioning here that the later age (91 days) compressive strength of all mixes was in the range between 68 and 74.8 MPa, which satisfies the minimum strength criteria in different standards for concrete to be considered as HSC [47,48,59,60].

Table 5 illustrates the comparison of compressive strength, tensile strength, and elastic modulus results of VA concrete with those of reference FA and CC. The results demonstrated a slightly high compressive strength of CC among all concrete specimens irrespective of age except at 91 days, where V20S10 exhibited the highest compressive strength. A slightly higher value of compressive strength of V20S10 concrete at later ages is due to its good pozzolanic nature and high fineness [30]. Moreover, it can be seen that the concretes containing VA demonstrated almost identical compressive strength results to that of corresponding FA concretes (V20 versus F20 and V20S10 versus F20S10) at 28 days. However, at the age of 7 days, V20 and F20S10 demonstrated a slightly high value of compressive strength as compared to F20 and V20S10 concretes and vice versa at the age of 91 days. The beneficial impact of adding EAFS along with V20 and F20 on strength development was noticeable at both early and later ages (7 and 91 days). This is due to the high fineness of EAFS, which improved the filling abilities and hydration reaction of cementitious composite.

As with compressive strength, CC demonstrated the highest splitting tensile strength (STS) among all concrete specimens irrespective of aging (Table 5). A slight reduction in STS for concretes containing VA or FA as compared to CC was obvious as there is always a direct relationship between the compressive and tensile properties of concrete. Although the tensile strength of VA concretes was lower than CC, it is evident that the STS of VA concrete mixes was comparable to or slightly higher than the corresponding FA concrete mixes (V20 versus F20 and V20S10 versus F20S10). Moreover, unlike compressive strength, there was no improvement in STS due to adding EAFS irrespective of aging or type of pozzolanic material (VA or FA) except at 28 days where the tensile strength of V20S10 was slightly high than that of V20 concrete. Therefore, despite the fact that there is always a direct relationship between compressive and STS, this was not validated for ternary mixes containing pozzolanic materials (VA or FA) and EAFS.

As presented in Table 5, the concrete mixes containing VA exhibited good stiffness potential. Both at early and at later ages (7 and 91 days), the elastic modulus of V20 concrete was comparable to or higher than CC, as well as reference F20 concrete. This is due to the improved filling ability of high-fineness VA at an early age and its continued pozzolanic reactivity at later ages. However, at 28 days, FA concrete with and without EAFS showed a higher elastic modulus as compared to corresponding VA concrete (V20 versus F20 and V20S10 versus F20S10). This is due to the relatively faster pozzolanic reaction rate of FA as compared to VA up to 28 days as evident from the development of other properties of FA concretes (compressive and tensile) at 28 days. As with STS, there was no improvement in elastic modulus due to adding EAFS into V20 or F20 concretes irrespective of aging except at 91 days where the elastic modulus of F20S10 was slightly higher than F20 concrete. Therefore, similar to tensile strength, a direct relationship between the elastic modulus of ternary concrete mixes (V20S10 or F20S10) and compressive strength was not validated.

### 3.3. Mechanical Properties of Concrete after Elevated Temperature Exposure

Table 6 shows the comparison of residual mechanical properties of VA concrete (V20 and V20S10) with those of CC and reference FA concrete under distinct elevated temperatures (200, 400, 600, and 800 °C). In order to effectively grasp the effect of increasing temperature, the ratio of each residual mechanical property was also calculated. The ratio was calculated by dividing the value of a residual mechanical property under elevated temperature by its corresponding value before exposure to elevated temperature. The specimens were exposed to elevated temperature after moist-curing for 91 days. The reason for curing concrete specimens up to 91 days was to allow the concrete mixes having pozzolanic materials to reach their maximum possible pozzolanic potential. This is evident from the test results presented in Table 5, showing that the difference between the mechanical properties of different concrete mixtures was lowest at the age of 91 days as compared to 7 and 28 days. A summary of the test results of different mechanical properties of all concrete mixes after their exposure to elevated temperatures is given in Table 6. The test results of each mechanical property presented in this table represent the average of three identical specimens.

#### 3.3.1. Effect of Elevated Temperature on Compressive Strength

**Figure 6:**
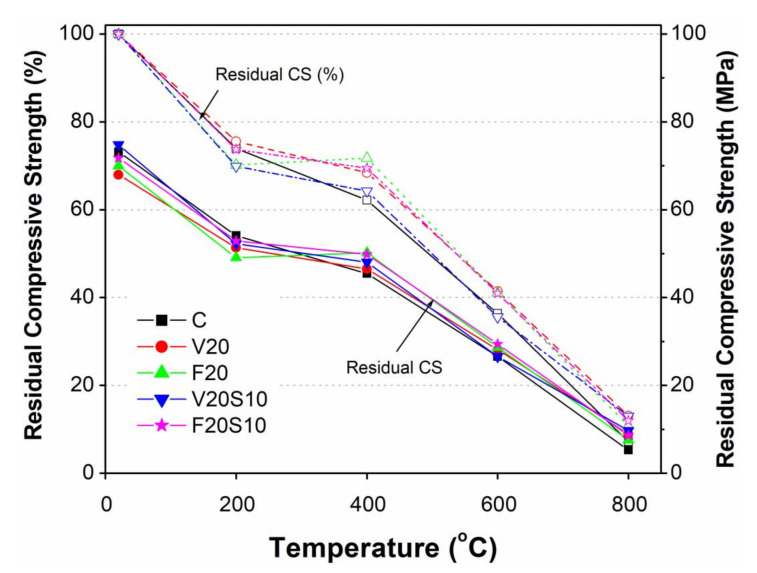
Comparison of residual compressive strength of VA concretes with that of CC and reference FA concretes.

It can be seen in Figure 6 that the CC specimen showed the highest value of RCS after exposure to 200 °C, followed by ternary (V20S10 and F20S10) and binary concrete specimens (V20 and F20). Despite the highest loss of free water (3.7%), CC possessed the highest RCS due to its relatively higher initial compressive strength among all mixes before exposure to elevated temperature. A comparison of RCS between V20 and F20 showed good potential of locally available VA in terms of its response under elevated temperature as compared to commercially used FA. Nonetheless, the loss of free water in V20 (2.8%) was slightly higher than that in F20 (2.5%), whereby V20 exhibited slightly higher RCS as compared to F20. As shown in Figure 6, the loss of RCS in V20 (24.4%) was lower than that in F20 concrete (29.8%). This could be due to the triggering of a late pozzolanic reaction in VA concrete under elevated temperature as described earlier.

Unlike at 200 °C, the current results demonstrated that all the concrete specimens containing pozzolanic materials as a partial substitute of cement (V20, F20, V20S10, F20S10) showed an encouraging response under moderate (400 °C) and high temperatures (600 and 800 °C). It is evident from Figure 6 that the RCS of concrete containing pozzolanic materials was always higher as compared to CC at all moderate and high temperatures. Moreover, it can also be seen in Figure 6 that, at these high temperatures, the RCS ratio of all concrete specimens containing pozzolanic materials was higher than that of the CC. Interestingly, as with the compressive strength at 91 days under normal curing, all concrete specimens containing EAFS exhibited a comparable or slightly higher compressive strength than their corresponding concrete mixture without EAFS (V20S10 versus V20 and F20S10 versus F20).

As shown in Figure 6, a slight drop in compressive strength (5 to 7%) was observed in concrete specimens containing pozzolanic materials upon increasing the temperature from 200 to 400 °C. Clearly, the drop in compressive strength in this heating regime was much lower as compared to the previous heating regime (20 to 200 °C), where it dropped by 24 to 30%. The drop in compressive strength for CC (12%) was noticeably more than all the concrete specimens containing pozzolanic materials. Thus, the current findings indicated significantly improved effects of adding pozzolanic materials (VA or FA) in offering high resistance to strength loss of concrete under moderate elevated temperature.

**Figure 7:**
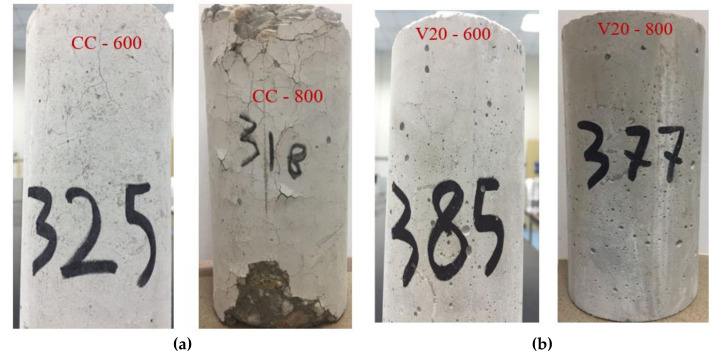

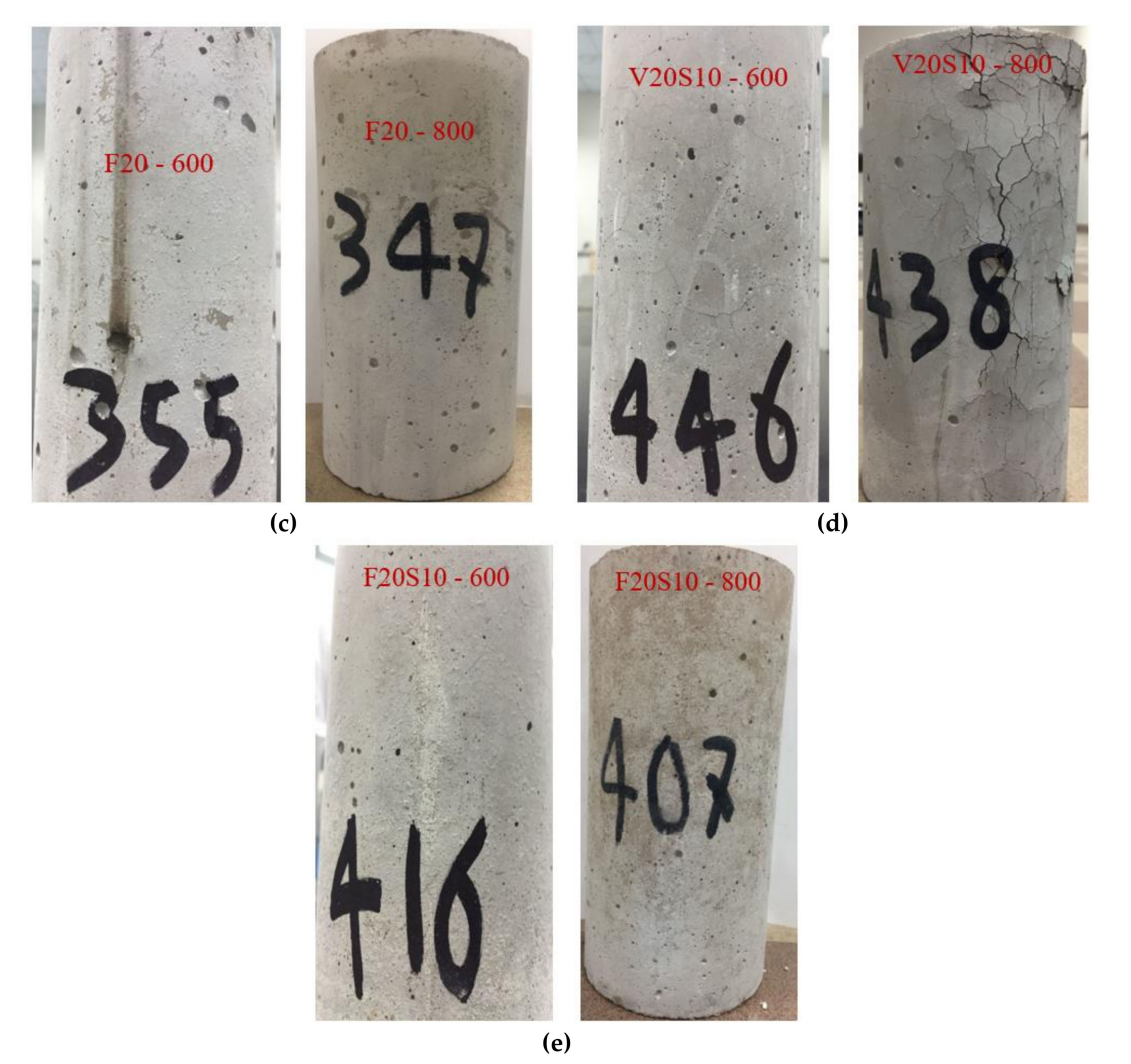
Comparison of surface cracks and spalling among some selected concrete specimens of all mixtures after exposure to 600 and 800 °C: (**a**) CC, (**b**) V20, (**c**) F20, (**d**) V20S10, and (**e**) F20S10.

Due to a further increase in temperature (400 to 600 °C), a significantly higher rate of loss of compressive strength occurred in all tested concrete specimens. The RCS reduced nearly to 40% for V20, F20, and F20S10. For CC and V20S10, it reduced even further to nearly 36%. After exposure to such a high temperature, the stability of concrete is seriously affected, making it very difficult to differentiate between the effective roles of different pozzolanic materials. According to past research [30], such a significant reduction in compressive strength is attributed to the decomposition of CSH and dehydration of Ca(OH)_2_ to free lime. Because of these changes, the volume of cementitious products increases, which ultimately leads to a reduction in cohesiveness within the paste matrix and the generation of hairline cracks within the concrete mass, as well as on its surface. Consequently, the crack in concrete cause a reduction in the compressive strength of CC, as well as for concrete containing pozzolanic materials. Figure 7 shows some selected samples for all concrete mixes with visible cracks after exposure to 600 °C. It is obvious that the relatively higher RCS values of concrete mixes containing pozzolanic materials than CC is due to their inherited ability to partly replace Ca(OH)_2_, thus offering a higher resistance to degradation at elevated temperature. According to Khurram et al. [30], these pozzolanas within the paste matrix form the refractory compounds of minerals and maintain significant strength even at high temperature.

With a further rise in temperature (600 to 800 °C), the trend of rapidly decreasing compressive strength continued in all concrete specimens in almost a similar manner to that when exposed to 600 °C. The rising temperature led to further physiochemical transformation and caused the re-crystallization of newly formed compounds. This phenomenon led to large numbers of cracks due to the rapid rate of expansion and shrinkage during heating and cooling phases, respectively. Similar to previous heating phases, the highest loss of compressive strength was observed in CC (Figure 6). In other words, all the tested concrete specimens containing pozzolanic materials possessed higher RCS as compared to CC. Figure 7 shows a comparison of the crack pattern after exposure to 600 and 800 °C. Although a clear surface spalling along with cracks was observed in specimens of CC and V20S10, the drop in RCS ratio remained almost the same (nearly 30%) among all tested concrete specimens (Figure 6), except for V20S10 (22%). Despite visible spalling on the surface of the V20S10 specimen, the observation of its relatively lower drop in RCS among all specimens is far from conventional understanding. In general, specimens with a higher number of cracks or surface spalling would lose more strength. No research has reported such incoherence in the past, and we believe that there is a need to explore this finding in the future research. Considering the outcome of the current experimental results, it may be safe to commend on the beneficial role of EAFS as an additive in concrete containing 20% VA. This may be because the presence of EAFS reduces the contents of free water and, thus, prevents the dehydration of the cementitious compound [30]. Furthermore, it can be seen in Figure 6 that, at such a high temperature, the concretes containing VA showed slightly higher RCS, as well as RCS ratio, compared to corresponding concretes containing FA (V20 versus F20 and V20S10 versus F20S10).

#### 3.3.2. Effect of Elevated Temperature on Splitting Tensile Strength

**Figure 8:**
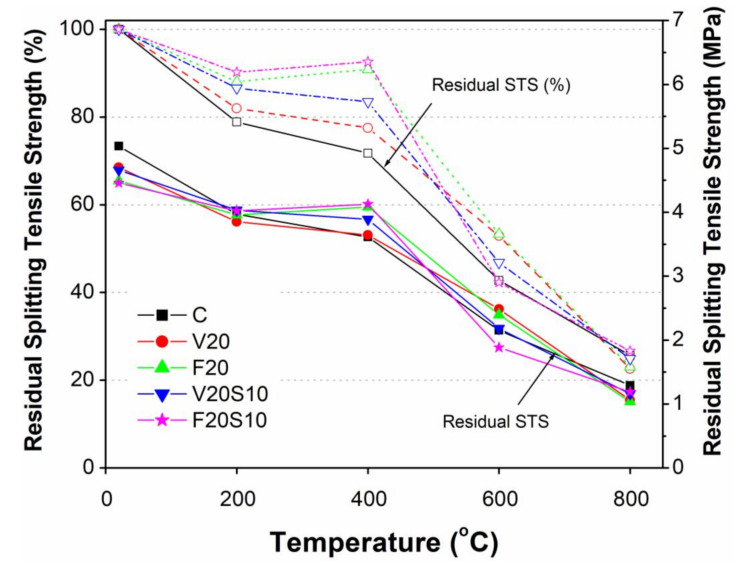
Comparison of residual splitting tensile strength of VA concretes with that of CC and reference FA concretes.

As shown in Figure 6 and Figure 8, the general trend of elevated temperature effects on the reduction in STS was similar to that of compressive strength. However, unlike compressive strength, the highest drop in splitting tensile strength (21%) due to increasing temperature from 20 to 200 °C occurred in CC (Figure 8). Moreover, the residual splitting tensile strength (RTS) of all concrete specimens exposed to 200 °C remained almost the same and a relatively lesser drop in STS (10 to 21%) was noted as compared to compressive strength (24 to 30%).

Similar to the compressive strength (5–12%), only a slight drop in STS occurred (3–7%) due to a further increase in temperature (200 to 400 °C). Although the highest drop of STS was noted in CC (7%), it was still quite lower than its respective drop in compressive strength (12%). Interestingly, there seemed to be no negative effects of elevating temperature (200 to 400 °C) on the STS of concrete specimens containing FA (F20 and F20S10), while only a slight drop in STS was observed for those containing VA (3 to 5%). In fact, the similar compressive and tensile behaviors of concrete are evidence of a direct relationship between them even under elevated temperatures. This fact e = was further noted for the specimens exposed to high elevated temperatures up to 800 °C. For instance, as with the compressive strength, a significant drop in STS occurred due to the exposure of specimens under 400 to 600 °C. As shown in Figure 6 and Figure 8, all concrete mixes (V20, F20, and V20S10) within this temperature range exhibited comparable or slightly higher RTS values than CC. Moreover, as with 400 °C exposure, a similar trend of RTS values and ratios for these concrete mixes to that of compressive strength was also observed. However, an exception was noted for F20S10 which, unlike compressive strength, resulted in the lowest RTS value among all tested concretes with an almost 50% drop in its RTS ratio (42.3% at 600 °C from 92.6% at 400 °C). As expected, the trend of decreasing STS continued in all concrete specimens due to the further increase of temperature from 600 to 800 °C. However, the loss of STS in this temperature range was significantly lower for all the concrete specimens (16 to 30%) as compared to the rapid loss (25 to 50%) that was being observed in the previous phase heating regime (400 to 600 °C). Moreover, unlike the RCS ratio (7 to 13%) of all the tested concretes, the RTS ratio (23 to 27%) was found to be slightly higher when the specimens were heated to a high temperature of 800 °C. This indicates the relatively lesser effects of severe heating temperature on the tensile properties of CC, as well as those containing pozzolanic materials with and without EAFS. However, as with RCS and its ratio, the values of RTS and its ratio were comparable among all tested concrete specimens.

A general trend of slightly higher STS was noted for specimens of ternary concretes mixes containing pozzolanic materials with EAFS as compared to their respective binary concretes (V20S10 versus V20 and F20S10 versus F20) irrespective of the exposed elevated temperatures. However, an exception was observed at 600 °C, and we are keen to explore the specific reason for this reduced STS of ternary concrete mixes as compared to their corresponding binary mixes in future research.

#### 3.3.3. Effect of Elevated Temperature on Elastic Modulus

**Figure 9:**
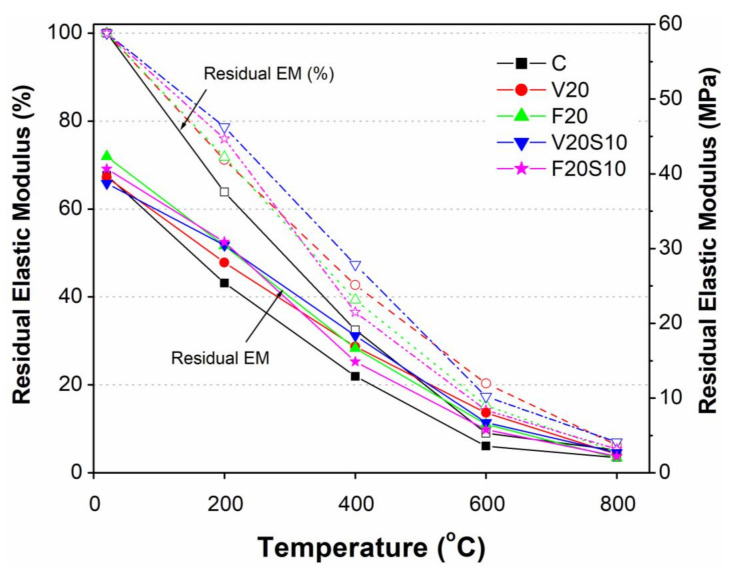
Comparison of residual elastic modulus of VA concretes with CC and reference FA concretes.

As with other mechanical properties, the elastic modulus of concrete decreased with increasing heating temperature. However, it was noted that the effects of increasing temperature on the reduction rates of elastic modulus were relatively faster compared to the respective compressive and splitting tensile strength of tested concrete specimens. As shown in Figure 6, Figure 8 and Figure 9, this is applicable particularly at low (20 to 200 °C) and moderately high temperatures (200 to 400 °C). For instance, the drop in elastic modulus due to increasing temperature from 20 to 200 °C of 21% to 36% was quite high compared to compressive (24 to 30%) and splitting tensile (10 to 21%) strengths. Accordingly, a further significant loss of stiffness was observed due to increasing temperature from 200 to 400 °C by almost 30% for all tested concretes, while there was no or only a slight loss of compressive (5 to 7%) and splitting tensile (3 to 5%) strengths observed in concretes containing pozzolanic materials. According to the current results, it may be concluded that a high percentage of concrete stiffness is already lost under low (20 to 200 °C) or moderately high temperature (200 to 400 °C). Therefore, the effect of high temperatures (beyond 400 and up to 800 °C) on the rates of decrease in elastic modulus is comparatively low. More specifically, the drop in elastic modulus for all tested concretes due to increasing temperature from 400 to 600 °C was as low as 22 to 30% as compared to 26 to 31% for compressive and 24% to 50% for slitting tensile strength, respectively. It reduced even further (4 to 14%) under 600 to 800 °C as compared to the high reduction in compressive (22 to 30%) and splitting tensile (17 to 30%) strengths.

As shown in Figure 9, although the elastic modulus of all tested concretes containing pozzolanic materials was quite comparable to that of CC, after heating the specimens to elevated temperatures, a clear difference in elastic modulus values could be noted among all tested concretes. Irrespective of the elevated temperatures, all concrete specimens containing pozzolanic materials exhibited higher residual elastic modulus (REM) and its ratio as compared to CC. This indicated relatively lesser effects of severe temperatures on the elastic modulus of concretes containing pozzolanic materials with and without EAFS. Moreover, as with the compressive and tensile strength properties, the ternary concretes containing pozzolanic materials along with EAFS showed slightly higher elastic modulus values as compared to corresponding binary concretes (V20S10 versus V20 and F20S10 versus F20) at almost all elevated temperatures except for 600 °C. Therefore, similar to the STS, we are keen to explore the reasons for this reduced elastic modulus of ternary concrete mixes as compared to the corresponding binary mixes in future studies.

### 3.4. Ultrasonic Pulse Velocity Before and After Exposure to Elevated Temperature

A summary of the ultrasonic pulse velocity (UPV) results of all tested specimens before and after exposure to elevated temperatures is presented in Table 5 and Table 6, respectively. The concrete specimens used in destructive compression tests were first used to perform a UPV test. According to ASTM C597, the UPV of all concrete specimens was calculated using the measured values of the travel time of pulse (microseconds) from the mass of cylindrical concrete specimens (travel distance = 0.2 m). All the values of UPV are reported as the average of three identical specimens. Before exposure to elevated temperatures, the ternary concrete specimens containing pozzolanic materials with EAFS (V20S10 and F20S10) exhibited slightly higher UPV values as compared to CC and the respective binary concrete mixes (V20 and F20). The higher values of UPV in ternary mixes can be attributed to the presence of relatively lesser voids due to fine particles of FA and VA in combination with EAFS. The current results of higher UPV values for ternary concrete mixes are consistent with their respective compressive strength results (Table 6). It can be noted that the UPV of binary concrete containing VA (V20) was slightly lower than that of CC and the reference binary concrete containing FA (F20). This indicates the presence of comparatively more air voids in V20 concrete. The above results are consistent with the strength results as the compressive strength of V20 concrete was slightly lower than that of the CC and F20 concrete.

**Figure 10:**
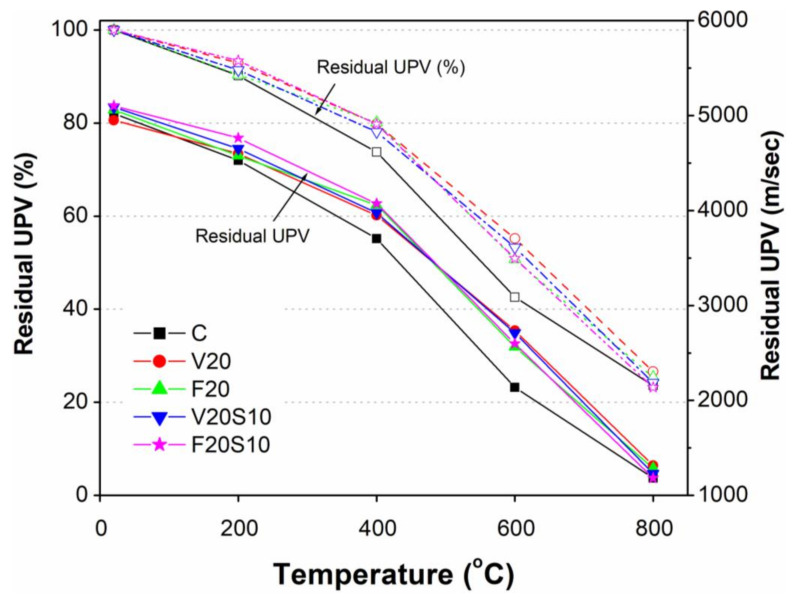
Comparison of residual UPV of VA concretes with those of CC and reference FA concretes.

As shown in Figure 10, the residual UPV of all concrete samples reduced with increasing exposure temperature. A gradual drop in UPV (up to 10%) was noted due to the initial increase in temperature from 20 to 200 °C. The highest drop in UPV observed for CC followed by F20, V20S10, V20, and F20S10. An almost similar trend was noticed for the weight loss of specimens due to the evaporation of free water. The higher amount of weight loss indicates the presence of a higher number of air voids in CC, which consequently led to lower UPV values. As compared to the previous heating regime, a relatively higher drop in UPV was noted upon increasing the exposure temperature from 200 to 400 °C. The highest drop in UPV among all tested concrete specimens was observed in CC (16%). The relatively lower drop in UPV for concretes containing pozzolanic materials is consistent with their respective compressive strength results. A lower drop in the UPV of concrete having pozzolanic materials indicates the presence of lesser internal air voids, which led to their higher RCS as compared to CC. With a further increase in exposure temperature from 400 to 600 °C, a sharp drop in UPV occurred for all concrete specimens, and the highest loss was observed for CC (31%). After CC, an almost identical high loss of UPV was noted in concrete specimens containing FA (30%) followed by those containing VA (25%). The reason for the sharp drop in UPV in this heating regime is attributed to the presence of large number of internal air voids. The number of internal voids increases with increasing dehydration of the binder due to rising temperature that consequently weakens the bond between binder and aggregates. Moreover, the decomposition of portlandite because of a chemical change in the binder leads to higher internal voids. As with UPV, similar trends of high weight loss and high loss of compressive strength were also observed for all concrete specimens. After exposure to elevated temperature from 600 to 800 °C, all concrete specimens showed a poor quality. This rapid loss of UPV values was a continuation of the previous heating regime, which was due to drastic changes in the microstructure and significant spalling of concrete at the specimen surface. The residual UPV of CC dropped to 1183 m/s, which was lowest among all tested concrete specimens. The residual UPV for binary (V20, F20) and ternary concrete (V20S10 and F20S10) specimens was recoded as 1315, 1286, 1230, and 1184 m/s, respectively. It is obvious that, with continuously rising temperature, the internal air voids in the concrete specimen continued increasing, resulting in a higher travel time of the pulse and, thus, lower values of residual UPV. Similar to all previous heating regimes, the trend of falling UPV of concrete specimens is consistent with their respective RCS results. Apparently, similar trends of residual UPV and RCS under rising exposure temperature suggest a good correlation between these properties of concrete specimens.

### 3.5. Relationship Between Residual Compressive Strength and UPV

**Figure 11:**
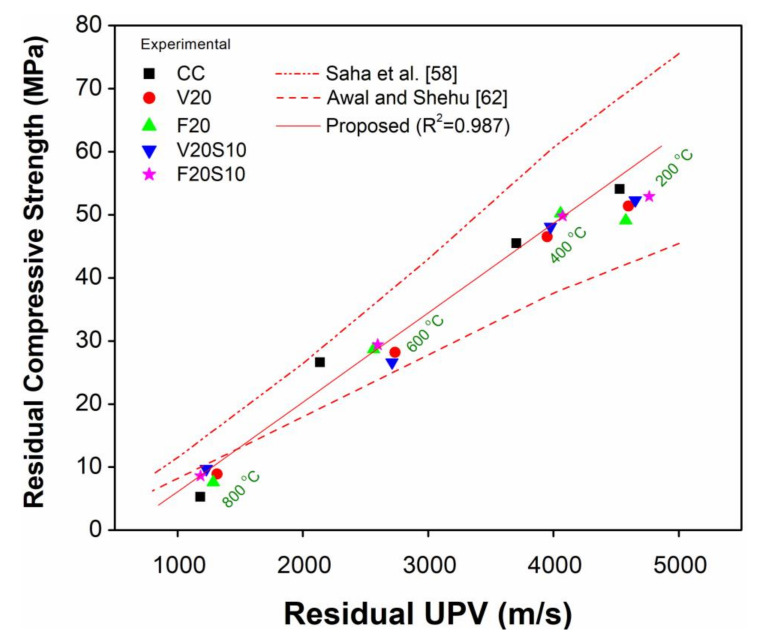
Relationship between residual compressive strength and UPV for control and concrete incorporating VA and FA.

Figure 11 shows the relationship between experimental results of RCS and residual UPV for all tested concrete specimens with respect to elevated temperature exposure. With increasing elevated temperature (200, 400, 600, and 800 °C), a general trend of decreased RCS with a decrease in residual UPV can be noted. Although not specifically for concrete containing VA, FA, or their blends with EAFS, a consistent relationship between RCS and UPV was reported by many researchers in the past [58,61,62,63]. Moreover, numerous researchers developed relationships between RCS and residual UPV of different types of concrete before and after exposure to elevated temperature [58,62,63]. The primary purpose of these relationships is to evaluate the RCS of concrete at any specified temperature by simply using the UPV without the need for conducting destructive compressive strength tests in the laboratory. Yang et al. [63] proposed a linear relationship between RCS ratio and RCS ratio of cylindrical concrete specimens (100 mm × 200 mm) of different mixture proportions (w/b ratio of 0.58 and 0.68) subjected to temperature ranging from 400 to 600 °C. However, to quantitatively evaluate the residual strength of fire-damaged concrete structures, their prediction equation requires UPV values before and after exposure to fire or elevated temperature. However, according to Awal and Shehu [62] and Saha et al. [58], the relationship between RCS and residual UPV is more convenient to assess the RCS of concrete only after exposure to fire or elevated temperature.

A comparison of current experimental data with the results of linear regression by Awal and Shehu for control concrete [62] and power regression by Saha et al. [58] is presented in Figure 11. Although, Awal and Shehu proposed different linear regressions (for control and palm oil fuel ash concretes) according to the exposure of 100 mm cube specimens up to 800 °C, their proposed regression for control concrete only reasonably predicts the residual strength of concrete for high exposure temperatures between 600 and 800 °C, while it underestimates the residual strength for exposure between 200 and 600 °C. The possible reason for this underestimated RCS can be due to the different geometry of their tested specimens (cubes), different heating regimes (exposure to peak temperature for 1 h only), and concrete of relatively lower strength (27–45 MPa) as compared to the current study (68–73 MPa). The power regression proposed by Saha et al. was based on exposure of cylindrical specimens (100 mm × 200 mm) of ferronickel and FA concrete up to 800 °C. However, unlike Awal and Shehu, their prediction equation overestimated residual strength of concrete. Despite a similar specimen geometry and heating regime, the reason for this overestimated RCS is because the Saha et al. model is based on a large range of experimental data (due to different types of concrete) as reflected by the very low coefficient of determination (*R*^2^ = 0.85). They also reported a high coefficient of variation (15%) for the prediction of RCS using their proposed power regression model [58]. Therefore, to develop a good correlation between experimental RCS and residual UPV values, a linear regression analysis was performed on the actual experimental data, and the following prediction equation is proposed:

R_CS_ = 0.0142 (R_UPV_) − 8.086 (1)

where R_CS_ is the residual compressive strength in MPa, and R_UPV_ is the residual ultrasonic pulse velocity in m/s. The coefficient of determination (*R*^2^) and the standard deviation for the above proposed equation are 0.987 and 4.24, respectively.

It can be seen from Figure 11 that the experimental data and the proposed regression line are very close to each other for almost all temperatures and up to an RCS of 50 MPa. However, a slight deviation of the regression line from experimental data can be observed for low exposure temperatures up to 200 °C. This is because, at low exposure temperature, the internal voids due to evaporation of free water caused a significant loss of strength (up to 30%) in HSC, while the loss of respective UPV values was low (up to 10%). The low loss of UPV at low exposure temperature is attributed to no or the existence of fewer cracks that would allow the pulse to travel in a lesser time. The reason for the good correlation between 400 and 800 °C is more discontinuity within the concrete due to a large number of internal voids and cracks, which resulted in greater UPV and strength loss with almost the same reduction rates. On the basis of the good correlation between experimental data and the regression line, it may be suggested to use the proposed equation to assess the RCS of fire-damaged (up to 800 °C) concrete incorporating VA, FA, and their blends with EAFS.

## 4. Conclusions

In this study, high-strength sustainable concretes using locally available volcanic ash were produced with and without incorporating EAFS. After determining the mechanical properties under normal curing, the concrete specimens were exposed to elevated temperatures up to 800 °C, and the influence of different heating regimes on the weight loss and mechanical properties of concrete were investigated. The values of UPV of concrete specimens before and after exposure to elevated temperatures were also measured and compared to the RCS of concrete. The following conclusions were made on the basis of this study:

1. Before exposure to elevated temperatures, HSC incorporating VA (V20 & V20S10) produced either comparable or slightly better results for all mechanical properties (compressive and splitting tensile strengths, elastic modulus, and UPV) to that of reference FA (F20 & F20S10) concrete, irrespective of aging. A slightly lower value of these mechanical properties was observed for the concretes containing pozzolanic materials (VA or FA) when compared to CC, particularly at early ages. This is attributed to the slow pozzolanic reactivity of these pozzolanic materials. However, due to a late pozzolanic reaction with aging, both VA and FA concretes possessed comparable results to that of CC at 91 days.
2. Looking at the encouraging response of VA concretes at 91 days, specimens were subjected to elevated temperatures, and gradual losses of weight, UPV, compressive strength, and tensile strength were noted for all tested concretes up to 400 °C, while significantly high rates of losses were observed under high-elevated temperatures between 400 and 800 °C. However, unlike other properties, a significantly high loss of elastic modulus was observed even at low elevated temperatures, due to the formation of internal air voids and hairline cracks because of the evaporation of free water at low temperatures and chemically bound water at high temperatures.
3. Up to 400 °C, the loss of compressive strength, tensile strength, elastic modulus, and UPV was in the range of 28–38%, 7–28%, 52–67%, and 20–24%, respectively. The highest losses were observed for CC, and a reasonably close agreement was noted between concretes containing VA and FA. The reason for lower losses among VA and FA concretes is their pozzolanic reactivity at relatively low elevated temperatures.
4. Between 400 and 600 °C, the respective losses of those properties were abruptly raised for all concretes to 58–64%, 47–58%, 80–81%, and 45–57%, with the highest loss for CC. At such a high temperature, the stability of concrete is seriously affected by the decomposition of CSH and dehydration of Ca(OH)_2_ to free lime. The relatively lesser loss among concretes with VA or FA than CC was due to their inherited ability to partly replace Ca(OH)_2_, thus offering a higher resistance to degradation at elevated temperatures.
5. Under high exposure temperature from 600 to 800 °C, the rate of high losses continued among all concretes, with values of 87–93%, 73–77%, 93–95%, and 75–77% for compressive strength, tensile strength, elastic modulus, and UPV, respectively. This is because of the large number of wider cracks and severe damage of concrete surfaces at such a high exposure temperature, irrespective of the type of concrete.
6. For all concretes, an almost uniform trend of effect of elevated temperature exposure on residual UPV and RCS was noted. Therefore, a linear regression analysis was performed between the residual UPV and RCS of tested concretes. On the basis of the good correlation between the experimental data and regression line, an equation was proposed to use UPV as a nondestructive test to assess the RCS of fire-damaged (up to 800 °C) concrete incorporating VA, FA, and their blends with EAFS.
7. The promising performance of concretes containing VA and EAFS before and after exposure to elevated temperature indicates that the use of these materials in construction as a partial substitute of cement can be very useful in terms of saving natural resources and protecting environment. However, it is suggested to extend this research to study the softening behavior of current concrete mixes at elevated temperature through their complete stress–strain curves. On this basis, a relationship between different concrete mechanical properties (strength and strains) can be proposed in terms of elevated temperature. In addition, other aspects of this research such as the influence of specimen dimension, heat conductivity, and heating/cooling rate on thermal damages need to be further explored.

## Figures and Tables

**Table 1:** Physical properties and chemical composition (oxides, % by weight) of cement, volcanic ash (VA), fly ash (FA), and electric arc furnace slag (EAFS).

	Cement	FA	VA	EAFS
	Physical properties			
Specific gravity (g/cm^3^)	3.15	2.83	2.64	3.69
Fineness (m^2^/kg) (Blain)	344	-	-	-
Fineness (m^2^/cc) (Microtrac S3500)	0.5670	1.027	0.431	0.589
	Chemical properties (oxides, % by weight)			
SiO_2_	20.9	51.5	46.4	16.1
Al_2_O_3_	5.18	24.3	14.4	3.80
Fe_2_O_3_ (SiO_2_ + Al_2_O_3_ + Fe_2_O_3_) *	3.04 -	8.87 84.7	12.8 73.6	31.7 51.6
CaO	63.9	5.15	8.80	30.6
MgO	1.65	3.50	8.30	9.84
Na_2_O	0.10	2.38	3.80	0.56
K_2_O	0.52	1.47	1.90	0.18
SO_3_	2.61	0.23	0.80	Less than 0.1
LOI **	2.51	0.25	2.80	No Ignitable
	Compounds (%)			
C_3_S	52.1	-	-	-
C_2_S	19.6	-	-	-
C_3_A	8.17	-	-	-
C_4_AF	8.81	-	-	-

* ASTM C618-15; ** LOI = loss on ignition.

**Table 2:** Particle size distribution of cement, VA, FA, and EAFS used in preparation of concrete.

Materials	Mean (µm)	Standard Deviation (µm)	D10 (µm)	D50 (µm)	D90 (µm)
Cement (C)	10.58	10.01	0.954	4.440	28.63
FA	5.840	4.000	0.694	1.819	13.59
VA	13.92	25.43	7.17	32.48	72.20
EAFS	10.85	25.13	1.130	24.13	130.5

**Table 3:** Physical properties of coarse and fine aggregates.

Materials	Apparent Specific Gravity	Bulk Specific Gravity (SSD)	Bulk Specific Gravity (OD)	Water Absorption Ratio (%)	Fineness Modulus
Coarse aggregate (20 mm)	2.708	2.695	2.687	0.272	-
Coarse aggregate (10 mm)	2.706	2.685	2.674	0.300	-
Fine aggregate	2.678	2.613	2.574	1.415	2.05

**Table 4:** Concrete mixture proportions and fresh properties.

Mix ID	w/b	a/b	s/a	Binder (kg/m^3^)				W (kg/m^3^)	Aggregates (kg/m^3^)			Admixture ASTM C-494 (Type F) % of Binder
				C	VA	FA	EAFS		FA	CA 10 mm	CA 20 mm	
CC	0.35	3.57	0.40	473	-	-	-	**166**	**670**	**370**	**650**	**1**
V20		3.57	0.40	378	95	-	-		670			1.2
F20		3.57	0.40	378	-	95	-		670			0.5
V20S10		3.47	0.38	378	95	-	47		623			1.2
F20S10		3.53	0.39	378	-	95	47		650			0.5
Fresh properties												
	Slump (mm)						Air content (%)			Unit weight (kg/m^3^)		
CC	90						3.5			2417		
V20	120						3.1			2378		
F20	150						2.3			2427		
V20S10	120						3.5			2450		
F20S10	100						3.1			2446		

**Table 5:** Mechanical properties of concrete with respect to aging before elevated temperature exposure and UPV.

Concrete Specimen ID	Compressive Strength (MPa)			Splitting Tensile Strength (MPa)			Elastic Modulus (GPa)			UPV (m/s)
	7 Days	28 Days	91 Days	7 Days	28 Days	91 Days	7 Days	28 Days	91 Days	91 Days
CC	42.6	51.4	73.1	3.96	4.93	5.04	36.0	39.5	41.6	5025
V20	36.8	49.2	68.0	3.72	4.28	4.57	37.7	36.7	41.5	5013
F20	35.6	49.5	70.0	3.42	4.20	4.64	34.7	42.0	39.1	5062
V20S10	37.4	46.5	74.8	3.39	4.51	4.65	32.4	33.8	40.1	5089
F20S10	40.9	46.9	71.8	3.20	3.81	4.43	33.4	35.4	40.6	5081

**Table 6:** Residual mechanical properties of concrete after exposure to elevated temperature.

Concrete Specimen ID	T (°C)	Residual Compressive Strength		Residual Splitting Tensile Strength		Residual Elastic Modulus		Residual UPV	
		MPa	Relative (%)	MPa	Relative (%)	GPa	Relative (%)	m/s	Relative (%)
CC	20	73.1 (3.6) *	100	5.03 (0.11)	100	39.8 (2.0)	100	5021 (7)	100
	200	54.4 (2.5)	74.0	3.97 (0.49)	78.9	25.4 (1.5)	63.8	4529 (67)	90.2
	400	45.5 (3.6)	62.2	3.61 (0.29)	71.8	12.9 (1.5)	32.5	3704 (19)	73.8
	600	26.6 (2.5)	36.4	2.15 (0.26)	42.8	3.6 (2.5)	9.0	2137 (46)	42.6
	800	5.3 (2.0)	7.2	1.29 (0.17)	25.7	2.1 (0.5)	5.2	1183 (14)	23.6
V20	20	68.0 (0.2)	100	4.70 (0.15)	100	39.5 (1.1)	100	4951 (56)	100
	200	51.4 (2.1)	75.6	3.85 (0.43)	82.0	28.1 (2.6)	71.2	4598 (85)	92.9
	400	46.5 (3.1)	68.4	3.64 (0.14)	77.5	16.9 (3.2)	42.7	3949 (103)	79.8
	600	28.2 (1.8)	41.5	2.49 (0.20)	52.9	8.0 (1.7)	20.3	2735 (67)	55.2
	800	8.9 (3.9)	13.1	1.06 (0.38)	22.6	2.5 (0.3)	6.3	1315 (21)	26.6
F20	20	70.0 (4.5)	100	4.49 (0.07)	100	42.3 (4.2)	100	5062 (176)	100
	200	49.1 (1.1)	70.2	3.96 (0.31)	88.1	30.4 (1.8)	71.8	4577 (110)	90.4
	400	50.2 (1.3)	71.7	4.08 (0.12)	90.9	16.7 (3.2)	39.4	4057 (102)	80.1
	600	28.7 (2.3)	41.0	2.40 (0.21)	53.3	6.5 (0.3)	15.3	2565 (100)	50.7
	800	7.6 (3.6)	10.8	1.04 (0.42)	23.1	2.0 (0.3)	4.7	1286 (90)	25.4
V20S10	20	74.8 (3.5)	100	4.66 (0.06)	100	38.7 (2.3)	100	5089 (145)	100
	200	52.3 (2.0)	69.9	4.03 (0.44)	86.6	30.5 (3.2)	78.7	4652 (87)	91.4
	400	48.1 (5.0)	64.3	3.89 (0.10)	83.5	18.3 (1.7)	47.4	3976 (77)	78.1
	600	26.6 (1.0)	35.6	2.18 (0.08)	46.8	6.7 (0.3)	17.4	2711 (87)	53.3
	800	9.7 (2.2)	13.0	1.16 (0.32)	24.9	2.7 (0.4)	7.0	1230 (48)	24.2
F20S10	20	71.8 (1.6)	100	4.46 (0.05)	100	40.7 (1.5)	100	5103 (67)	100
	200	52.9 (4.1)	73.7	4.02 (0.25)	90.2	30.9 (2.1)	76.0	4764 (120)	93.4
	400	49.8 (2.3)	69.4	4.13 (0.20)	92.6	14.9 (2.5)	36.6	4073 (87)	79.8
	600	29.4 (2.5)	41.0	1.88 (0.16)	42.3	5.8 (0.2)	14.2	2598 (86)	50.9
	800	8.6 (3.8)	12.0	1.19 (0.35)	26.7	2.2 (0.5)	5.4	1184 (62)	23.2

* Values in parentheses indicate the standard deviation of all test results.

## Data Availability

The data presented in this study are available on request from the corresponding author.
